# Effects of different types of neonatal pain on somatosensory and cognitive development in male juvenile rats

**DOI:** 10.1002/brb3.3309

**Published:** 2023-11-15

**Authors:** Ru Ling, Yueshu Wang, Wen Zheng, Cuiting Min, Mengying Chen, Dongqing Xia, Xiaonan Li

**Affiliations:** ^1^ Department of Child Health Care Children's Hospital of Nanjing Medical University Nanjing Jiangsu P. R. China

**Keywords:** cognition, inflammatory pain, needling pain, neonatal, somatosensory

## Abstract

**Background:**

Premature infants are inevitably exposed to painful events, including repetitive procedures, inflammation, or mixed stimulation that may induce long‐term behavioral outcomes. Here, we set up three neonatal painful models to investigate their long‐term effect on somatosensation and cognition.

**Methods:**

Three types of neonatal pain models in rat were set up. Rat pups were randomly assigned to four groups. The needling pain (NP) group received repetitive needle pricks on the paws from the day of birth (PD0) to postnatal day 7 (PD7) to mimic the diagnostic and therapeutic procedures. The inflammatory pain (IP) group received the injection of carrageenan into the left hindpaw at PD3 to induce IP in peripheral tissues. The mixed pain group received a combination of the NP and IP (NIP). The control (CON) group was untreated. We performed behavioral and biochemical testing of juvenile rats (PD21–PD26).

**Results:**

The NIP group showed a longer hypersensitivity than the NP group, when given a secondary inflammatory stimulation. NP led to insensitivity to anxiety‐causing stimuli and impairment of fear memory both aggravated by NIP. NP reduced the expression of synapse‐related molecules (GluN1/PSD95/GFAP) in the medial prefrontal cortex, and NIP exacerbated this decrease. The corticosterone secretion in the NIP group increased after the behavioral task, compared with those in other three groups.

**Conclusion:**

A combination of NP with inflammation occurring in the neonatal period might aggravate the adverse effects of each on somatosensory and cognitive development of rats, the mechanism of which might be associated with the increase of corticosterone secretion and the dysregulation of synaptic molecules.

## Graphical abstract

A combination of needling and inflammatory pain exerts more serious effect on neonatesâ€™ neurodevelopment through increasing the pain hypersensitivity, impairing conditional memory, inducing the insensitivity to anxiety‐causing stimuli, and damaging synaptic function in the mPFC.

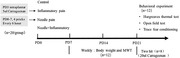

## 1 INTRODUCTION

New medical technologies have raised the survival rate of premature infants (Zhang et al., 2021). However, due to the immature development and complications, preterm infants often require long‐term hospitalization in the neonatal intensive care unit (NICU), during which they will inevitably experience painful procedures, including heel lances, vascular puncture, tracheal intubation, surgical incision, and so on. Previous data have shown that each infant undergoes a mean of 10 invasive procedures a day during hospitalization in the NICU (Roofthooft et al., 2014), represented by venepunctures and heel lances (Carbajal et al., 2008; Stevens et al., 2003). Besides, these procedures not only involve considerable pain during their application but are also frequently followed by local inflammation accompanied by hyperalgesia and pain lasting for several hours or 7 days (Anand, 1998).

During early postnatal period, the central nervous system (CNS) develops fast, but this development is susceptible to adverse stimuli (Semple et al., 2013). Preterm infants can feel pain (Fitzgerald, 1995), so painful stimuli may retard their CNS development. Pain from multiple repetitive procedures in the neonatal period, if not relieved immediately, has long‐term adverse effects on neurodevelopment (Duerden et al., 2022; Gaspardo et al., 2018; Schneider et al., 2018; Vinall et al., 2014) and pain sensitivity in childhood and adulthood (Anseloni et al., 2002; Hermann et al., 2006; Taddio et al., 2009).

The animal model of neonatal pain is commonly established with needling that simulates invasive needle‐related procedures, or injection that induces peripheral inflammatory exudation. It has been confirmed that neonatal repetitive needling pricks can increase the pain sensitivity (Anand et al., 1999; Ruda et al., 2000) and impair memory (Chen et al., 2016). Early inflammatory pain (IP) can also cause neurodegenerative changes in the brain (Dührsen et al., 2013) and increase pain sensitivity after the second stimuli (Ruda et al., 2000; Tachibana et al., 2001). Most previous studies have only focused on the adverse effect of a single type of pain, which refers to giving only one type of stimulus in the early postnatal period with no attention paid to the integrated effects of different types of pain. However, preterm infants usually experience both procedural and IP at the same time in the clinical environment; it is not clear whether mixed pain leads to more serious effects.

In the neonates, pain dysregulates the development of the CNS through various pathways (Schwaller & Fitzgerald, 2014). The medial prefrontal cortex (mPFC) is involved in pain processing and cognitive function (Huang et al., 2019; Klune et al., 2021), which depends on normal synaptic function. Synapses are constantly pruned in the brain of infants, and early‐life stressors disturb synaptic formation and pruning (Khundrakpam et al., 2016), further affecting brain and cognitive development. Postsynaptic density protein 95 (PSD95) is a vital synaptic marker that maintains the synaptic structure and function (Fitzgerald et al., 2015). As a scaffolding protein located at excitatory synapses, PSD95 is involved in the stabilization, recruitment, and trafficking of *N*‐methyl‐d‐aspartic acid receptors to the postsynaptic membrane (Coley & Gao, 2018). Glutamate‐gated NMDA receptor 1 (GluN1) is essential in excitatory synaptic transmission and activated significantly via pain‐related pathways (Ji et al., 2003). Previous studies have revealed that neonatal pain alters neuronal excitability (Anand et al., 2007) and glutamatergic function (Mooney‐Leber et al., 2018). In addition, recent studies have found that astrocytes take part in the development of synapses and glutamate signaling, which involves glial fibrillary acidic protein (GFAP) specific in astrocytes (Verkhratsky & Chvátal, 2020; Verkhratsky & Nedergaard, 2014). The role of GFAP in the effect of neonatal pain has not been studied yet.

Therefore, this study verified whether repeated procedural pain and IP simultaneously during early life could exert a worse effect on later somatosensory and cognitive development and teased out related mechanisms.

## 2 METHODS

### 2.1 Animals

All the experiments performed on rats were approved by the Ethics Committee of Animal Experiments of Nanjing Medical University (NJMU/IACUC2105028). Efforts were made to minimize animal suffering and reduce the number of animals by using the same animals in multiple behavioral experiments and observing molecular expression in rats after tasks.

All rats were born on the 21st or 22nd day of gestation from time‐pregnant Sprague–Dawley dams which were purchased from Vital River Laboratories and housed individually in the standard environment at 22 ± 2°C under a 12 h light/dark cycle (lights on at 7:00 am), with food and water available ad libitum.

### 2.2 Experimental design

For rat pups, the birth day was set as postnatal day 0 (PD0). At PD0, only male pups were selected, in order to rule out sex and nutrition‐related confounding factors. A total of 80 Sprague–Dawley male rat pups were included, and randomized into four groups exposed to: (1) needling pain (NP) group, (2) IP group, (3) a combination of needling and inflammatory pain (NIP) group, (4) no pain (CON) group. Male pups were standardized to *N* = 10 per dam, and individual identifications were made by labeling numbers on their backs on PD0. Then they were randomly assigned into four groups to receive different neonatal treatments and were adopted by a step‐mother.

**FIGURE 1:**
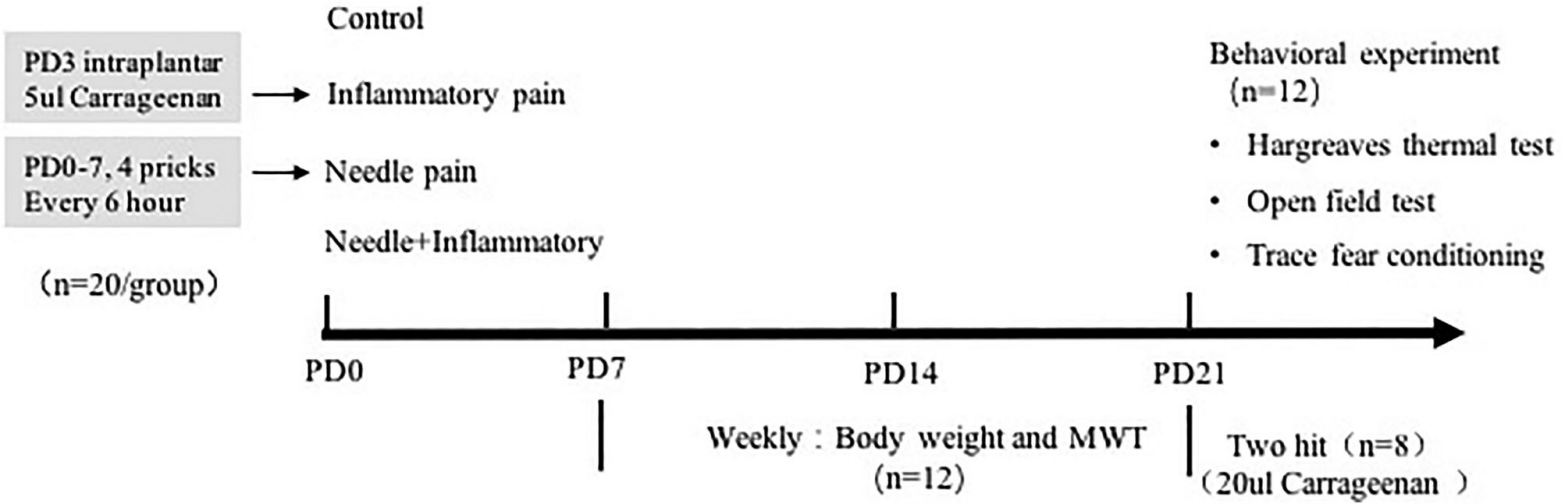
Experimental protocol. MWTs, mechanical withdrawal thresholds.

Weight and mechanical sensitivity were tested at the age of PD7, PD14, and PD21. Other types of behavioral testing, including thermal sensitivity test, open field test (OFT) and trace fear conditioning test, were conducted from the time of PD21. The experimental protocol is shown in Figure 1.

### 2.3 Painful treatment

#### 2.3.1 NP

According to our previous study (Chen et al., 2016), with some adjustment to the study of Anand et al. (1999), a repetitive NP model was established to mimic the experience of preterm neonates exposed to painful procedures in the NICU. From PD0–PD7, the pups were pricked using a sterile 28‐gauge lancet (Accu‐Chek Softclix, Roche) four times daily with an interval of 6 h. In turns, the four sites were pricks (from left to right, hindpaw to forepaw), in order that a previous model with 10 pricks did not make a difference in the mechanical sensitivity from four pricks (van den Hoogen et al., 2020), and that single‐foot stimulation four times per day in the neonatal period did not affect baseline mechanical sensitivity but increased sensitivity after re‐incision in adulthood (Knaepen et al., 2013).

#### 2.3.2 IP

According to the study of Victoria et al. (2013b), IP was induced through injecting 5 μL of carrageenan with the concentration of 1% (dissolved in saline, Sigma) into the mid‐plantar surface of the left hindpaw at PD3. Intraplantar saline control was not used, as a previous study confirmed that this operation would induce inflammation persisting for 24 h (Victoria et al., 2013b).

#### 2.3.3 NIP

NIP was induced through needling four times per day (every 6 h) from PD0 to PD7, plus a single injection of carrageenan on PD3 on the left hindpaw at the same day.

### 2.4 Behavioral testing

At different time points, a single blind experiment was conducted by an experienced observer. Weight and mechanical withdrawal threshold (MWT) were tested at the age of PD7, PD14, and PD21. At PD21, eight rats were chosen from each group for continuous mechanical sensitivity testing after secondary inflammatory stimulation. The rest rats were subjected to behavioral testing, including hargreaves thermal test, OFT, and trace fear conditioning, which were conducted from the time of PD22 (*n* = 12).

#### 2.4.1 von Frey test

Calibrated filaments (Stoelting Co) were used to measure the MWT on PD7, PD14, and PD21 on the left hindpaw. Each rat had stayed individually on a wire mesh platform covered with a transparent acrylic cage for a 30‐min acclimatization before the experiment. The ascending stimulus method was used (Deuis et al., 2017). The filament with a low bending force (0.4 g) was used first to put pressure vertically on the plantar surface. A positive action was considered a quick plantar withdrawal, flinching or licking at the time of or immediately after each stimulation. The operation was repeated for five times, and once the reaction was positive for at least three times in the five times, the final force was recorded as the MWT. Otherwise, a higher level of filament was used to continue the experiments. At the age of PD21, partial animals from the four group (*n* = 8) received the induction of IP. Carrageenan (20 μL, 1% dissolved in saline, Sigma) was subcutaneously injected with an insulin‐type syringe (30G needle) into the mid‐plantar surface of the left hindpaw. The MWT of the left hind paw was measured at 1, 24, 48, and 72 h after stimulation.

#### 2.4.2 Hargreaves thermal test

All rat pup groups were tested for paw withdrawal latency (PWL) using a hot plate (IITC) to assess the thermal pain threshold at PD22. The hot plate comprised a 25 cm × 25 cm metal plate with a fitted glass cage. Before the experiment, each rat was conditioned on the unheated plate for 10 min. Based on previous research (Nuseir et al., 2019), the hot plate was set to 52°C with a cut‐off time of 20 s to prevent injury to the rats. The time from entering the hot plate to the appearance of foot retreat or paw licking was recorded as PWL. The experiment was repeated for three times with an interval of 15 min, and the results were average.

#### 2.4.3 Open field test (OFT)

OFT was used to assess locomotor activity and anxiety‐like behavior at PD23. This arena consisted of a black square plastic floor (50 cm × 50 cm) surrounded by a 50 cm wall and was divided into the central region and the peripheral region, each with an area of 12,500 cm^2^. One rat was gently placed in the center of the field and allowed to face the same direction, move freely for 10 min. The behavior of the rat was tracked automatically using a video tracking system (Top View Animal Behavior Analyzing System; Clever Sys., Inc.). Rats in each group were tested in turn. The arena was disinfected with 75% ethanol and was not used until the smell disappeared. The following indexes were recorded: total distance (actual locomotor path in the whole area), inner time (time spent in the central area), inner distance (actual locomotor paths in the central are divided by total distance), and speed in inner area.

#### 2.4.4 Trace fear conditioning (trace FC) test

The experiment was performed from PD24 to PD26. The equipment was cleaned with 75% ethanol between trials. On the first day, each rat was acclimated in a training box for 12 min. The next day, the rat was allowed to explore the same box for 3 min and then to experience four consecutive training blocks. Each training block consisted of a 20‐s 1‐kHz 80‐dB tone (conditioned stimulus, CS), followed by an 18‐s trace interval and a 2‐s 1.0‐mA foot shock (unconditioned stimulus, US) before a 40‐s intertrial interval (ITI). On the third day, the rat's performance in the training box was recorded for 3 min to assess fear memories associated with environmental condition. Then, the rat was put into a novel chamber to test its conditioned memories of the tone (CS) associated with shock (US) in the absence of contextual cues. After adaptation for 3 min, the rat was tested with two blocks consisting of a 20‐s 1‐kHz 80‐dB tone, a 20‐s trace interval of silence and a 40‐s ITI. The experimental protocol is shown in Figure 3a. The automatic motion detection software (FreezeFrame, Coulbourn Instruments) was used to record rat behaviors during each test and calculate the percentage of freezing time, reflecting the memory of fearful events.

### 2.5 Reverse transcription quantitative real‐time PCR (RT‐qPCR)

On PD26, at 30 min after the experiment of trace FC, the rat was sacrificed under anesthesia. The brain was immediately removed out of the skull, and the whole mPFC tissue was quickly separated and was halved with one half going to PCR and the other for WB. The tissues were placed onto ice and stored in 2.0 mL microcentrifugation tube at −80°C until used for PCR or WB analyses. Total RNA was extracted from half of each tissue homogenate (*n* = 8 per group) using TRIzol (Invitrogen). RNA concentration was evaluated by NanoDrop 1000 spectrophotometer (Thermo), and the integrity of total RNA was evaluated by agarose gel electrophoresis. Total cDNA in 1.0 μg of RNA sample was synthesized using a Transcriptor First Strand cDNA Synthesis Kit (Roche Diagnostics GmbH), as described by the manufacturer. PCR amplification of a subset of the cDNA samples using glyceraldehyde‐3‐phosphate dehydrogenase (GAPDH) primers was performed to confirm successful reverse transcription.

RT‐PCR was performed using the SYBR GREEN (Roche Diagnostics GmbH) real‐time PCR system (ABI‐7500) with the following cycles: 50°C for 2 min, 95°C for 10 min, 40 cycles of 95°C for 15 s, and 60°C for 1 min. The mRNA levels were normalized to those of corresponding GAPDH mRNA levels. Data were analyzed with the 2^−△△^ *^Ct^* method. The primer sequences are listed in Table 1.

### 2.6 ELISA

On PD26, at 30 min after the last behavioral experiment, peripheral blood was sampled, and the upper serum was centrifuged. Serum corticosterone concentrations were measured by using the enzyme‐linked immunosorbent assay (ELISA) kit (CUSABIO) according to the manufacturer's protocol (*n* = 8 per group).

### 2.7 Western blotting

The total protein was extracted from half of each tissue homogenate of the whole mPFC (*n* = 3 per group) using ice‐cold lysis buffer (Beyotime Inc.), which contained 20 mM Tris, pH 7.5, 150 mM NaCl, 1% Triton X‐100, 2.5 mM sodium pyrophosphate, 1 mM EDTA, 1% Na_3_VO_4_, 0.5 μg/mL leupeptin, and 1 mM phenylmethanesulfonyl fluoride. After centrifugation at 12,000 × *g* for 15 min, the supernatant was collected. The protein concentration was determined using the BCA protein assay kit (Thermo Fisher Scientific). An equal amount of total protein was loaded in each lane, and then the proteins were separated by 8%−12% sodium dodecyl sulfate‐polyacrylamide gel electrophoresis transferred onto PVDF membranes (0.45 μm, Millipore), blocked with Tris‐buffered saline and 0.1% Tween 20 (TBST) plus 5% fat‐free milk for 1 h at room temperature and incubated with primary antibodies overnight at 4°C. The antibodies included anti‐GluN1 (Abcam, ab109182, 1:2000), anti‐PSD95 (Abcam, ab238135, 1:2000), anti‐GFAP (Affinity Biosciences, DF6040, 1:500), and anti‐GAPDH (ProteinTech, 60004‐1‐Ig, 1:2000) antibodies. Following five rounds of washing with PBST (6 min each), the membranes were incubated with goat anti‐rabbit IgG (1:2000, Beyotime Inc.) at room temperature for 1 h and then washed again with TBST (five rounds, 6 min each). The target bands were visualized by chemiluminescence (ECL) detection reagent (Amersham, Buckinghamshire) and analyzed by a ChemiDoc XRS + Imaging System (Bio‐Rad Molecular Image). When reprobing was necessary, the membrane with blot was incubated with stripping buffer (Beyotime Inc.) for 15  min at room temperature. Then the membrane was blocked with TBST plus 5% fat‐free milk for 1 h and incubated with another primary antibody overnight at 4°C, followed by subsequent steps as above. The results were expressed as the ratio of the intensity of target protein to that of GAPDH from the same membrane.

### 2.8 Statistics analysis

All data in this study were expressed as the mean ± SEM. Behavioral outcomes and molecular expression among four groups were analyzed by one‐way ANOVA followed by Tukey post hoc comparisons for assessing pairwise differences. Differences in the paw thickness, MWT, trace FC training, and testing phase data among four groups were analyzed using two‐way ANOVA with time and treatment as variables, followed by Tukey post hoc comparisons for assessing pairwise differences. All statistical analyses were performed using IBM SPSS 22.0, and statistical significance was set at *p* < .05.

## 3 RESULTS

### 3.1 Effect of neonatal pain on body weight of rat pups

Body weight was measured weekly from birth to PD21 (Table 2). No differences were observed between the four groups in body weight at PD0 (*p* > .05) and PD21 (*p* > .05). There was a significant difference in body weight among the four groups at PD7 (*F*[3, 48] = 35.81, *p* < .001), and post hoc analysis suggested that the body weights in the IP and NP groups were lower than that in the CON group (*p* < .001), whereas the weight in the NIP group was significantly lower than all three groups (*p* < .01). At PD15, there was still a difference in body weight between the four groups (*F*[3, 48] = 5.11, *p* < .01), and post hoc analysis showed that only the body weight in the NIP groups was significantly lower than that in the CON group (*p* < .01).

**TABLE 1:** Body weight during the study period.

Age	CON	IP	NP	NIP
PD0	6.54 ± 0.30	6.27 ± 0.54	6.58 ± 0.39	7.07 ± 0.93
PD7	22.33 ± 0.92	18.36 ± 1.66^*^	18.60 ± 2.34^*^	15.93 ± 1.02^*^,^**^
PD14	34.64 ± 1.50	33.46 ± 1.24	33.43 ± 1.65	32.03 ± 2.26^*^
PD21	53.18 ± 3.92	52.43 ± 3.04	53.05 ± 2.49	53.39 ± 2.23

*Note*: Data show mean weight gain during study period. One‐way ANOVA with Bonferroni post hoc test was used to compare the body weights in each stage. Data are expressed as the mean ± SEM.

Abbreviations: CON, control; IP, inflammatory pain; NIP, needling and inflammatory pain; NP, needling pain.

^*^ *p* < .05, compared to the CON group.

^**^ *p* < .05, compared to the NP and IP group, one‐way ANOVA with Tukey post hoc comparison at each age, *n* = 12 per group.

**TABLE 2:** Primer sequences used for mRNA quantification by real‐time PCR (RT‐PCR).

	Forward primer 5′–3′	Reverse primer 5′–3′
GluN1	CGGCTCTTGGAAGATACAGC	GTGAAGTGGTCGTTGGGAGT
PSD95	TCATAACTCCCCATGCCATT	CTCATGCAAACCAGCAAAGA
GFAP	CCAAGATGAAACCAACCT	CGCTGTGAGGTCTGGCTT
GAPDH	GGCTCTCTGCTCCTCCCTGTTCTA	CGTCCGATACGGCCAAATCCGT

Abbreviations: GAPDH, glyceraldehyde‐3‐phosphate dehydrogenase; GFAP, glial fibrillary acidic protein; PSD95, postsynaptic density protein 95.

### 3.2 Carrageenan injection caused swelling of the hindpaw

**FIGURE 2:**
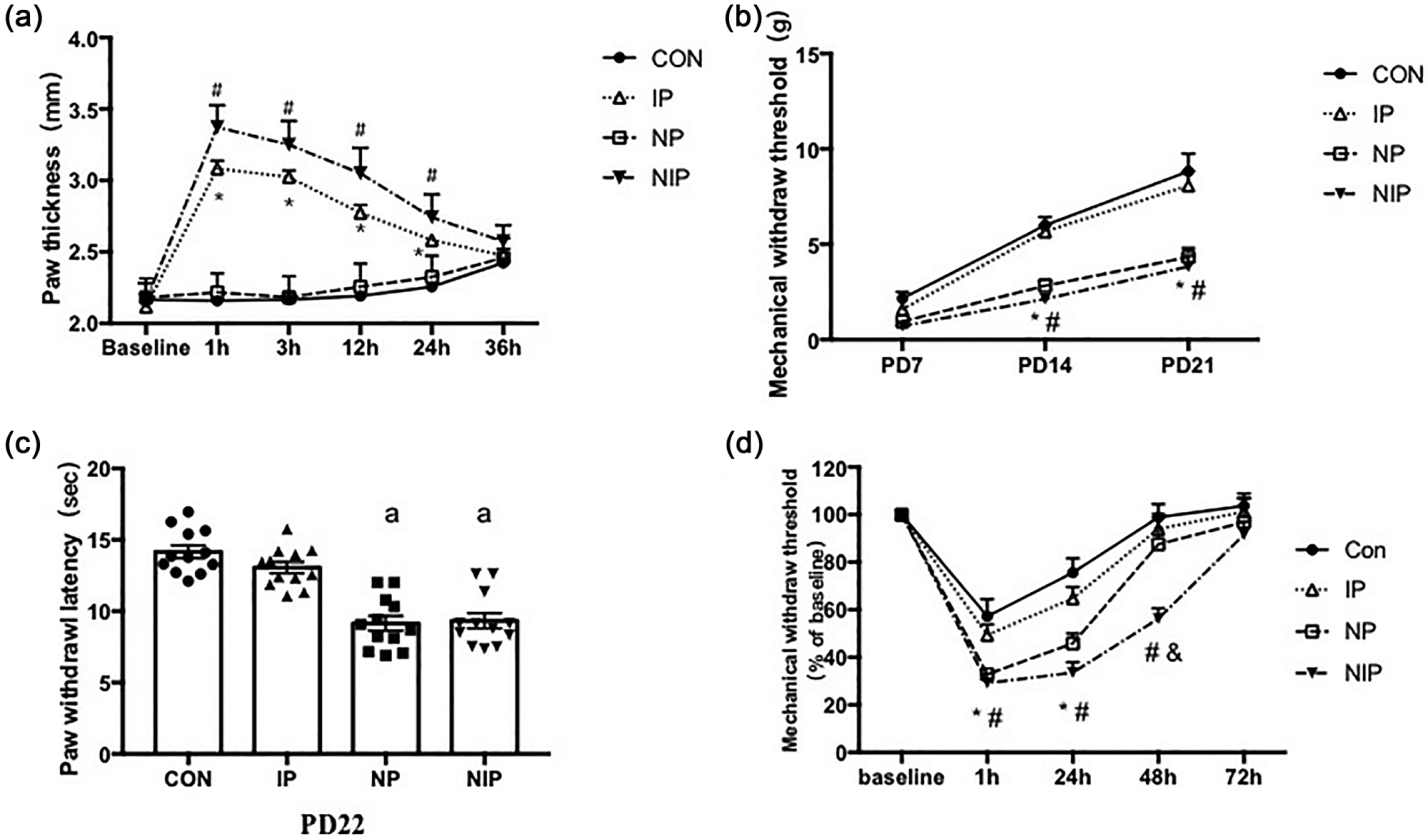
(a) Left hindpaw thickness measurements of neonatal rat pups following injection of carrageenan at PD3. Data are shown as mean ± SEM. ^*^ *p* < .05, compared with the group of control (CON) and needling pain (NP). ^#^ *p* < .05, compared with the group of CON, NP and inflammatory pain (IP), two‐way ANOVA with Tukey post hoc comparison. *n* = 12 per group. (b) Mechanical withdrawal thresholds (MWTs) of the left hindpaw from PD7 to PD21 among the four groups pup. Data are shown as mean ± SEM. ^*^The MWT in NP group was lower than that of CON and IP group, *p* < .05. ^#^The MWT in needling and inflammatory pain (NIP) group was lower than that of CON and IP group, *p* < .05, two‐way ANOVA with Tukey post hoc comparison. *n* = 12 per group. (c) Thermal stimulation sensitivity test of the left hindpaw at PD21. Data are shown as mean ± SEM. ^a^ *p* < .05, compared with the group of CON and IP, one‐way ANOVA with Tukey post hoc comparison. *n* = 12 per group. (d) MWT of the left hindpaw at 1, 24, 48, and 72 h after an inflammatory stimulation at PD42. ^*^The MWT in the NP group was lower than those in the CON and IP groups, *p* < .05. ^#^The MWT in NIP group was lower than those in the CON and IP group, *p* < .05. ^&^The MWT in NIP group was lower than that in the NP group, *p* < .05, two‐way ANOVA with Tukey post hoc comparison. Data are shown as mean ± SEM, *n* = 8 per group.

As shown in Figure 2a, the paw thickness increased significantly at 1, 3, 12, and 24 h after injection of carrageenan in IP and NIP groups (*p* < .05). The effect of pain was obvious (*F*[3, 264] = 307.2, *p* < .0001), and a significant interaction with time (*F*[15, 264] = 37.47, *p* < .0001). NP did not increase paw thickness at the same time points, compared with that in the CON group (*p* > .05), and there was still no difference in paw thickness between NP and CON groups at PD7 (Supporting Information 1). Moreover, the NIP group showed thicker hindpaw than the IP group at 1, 3, 12, and 24 h after carrageenan injection (*p* < .001).

### 3.3 NP and mixed pain increased pain sensitivity

Mechanical sensitivity of the left hindpaw was measured at PD7, PD14, and PD21. As shown in Figure 2b, the MWT increased significantly on 14 and PD21 in NP and NIP groups (*p* < .05). The effect of pain was obvious (*F*[3, 132] = 39.44, *p* < .0001) and changed significantly with time (*F*[6, 132] = 4.40, *p* < .001). However, there was no significant difference in MWT between NP and NIP groups (*p* > .05) at every observation site (PD7, PD14, and PD21).

Thermal stimulation sensitivity was tested at PD22, as shown in Figure 2c. A significant difference in PWL was observed among the four groups (*F*[3, 44] = 28.71, *p* < .0001). The NP and NIP groups showed lower PWL than CON or IP groups (*p* < .05). No differences were observed between the NP and NIP groups (*p* > .05).

We also observed mechanical sensitivity at 1, 24, 48, and 72 h after the second carrageenan injection at PD21. As shown in Figure 2d, the threshold for withdrawal from the von Frey mechanical stimulus decreased below the baseline level at 1 and 24 h following carrageenan injection in CON group (*p* < .05), which returned to the baseline level after 48 h of stimulation (*p* > .05). The effect of pain on MWT was significant (*F*[3, 140] = 20.29, *p* < .0001) and changed with time (*F*[12, 140] = 2.75, *p* < .01). The MWT decreased significantly at 1 and 24 h after carrageenan injection in the NP and NIP groups (*p* < .05). Moreover, the MWT in the NIP group was still lower than those of the CON, IP, or NP groups at 48 h following a second carrageenan stimulation (NIP vs. CON, IP or NP, *p* < .001). At 72 h, the MWT values in all groups returned to the baseline level, with no difference among groups.

### 3.4 Mixed pain increases insensitivity to anxiety‐causing stimuli

**FIGURE 3:**
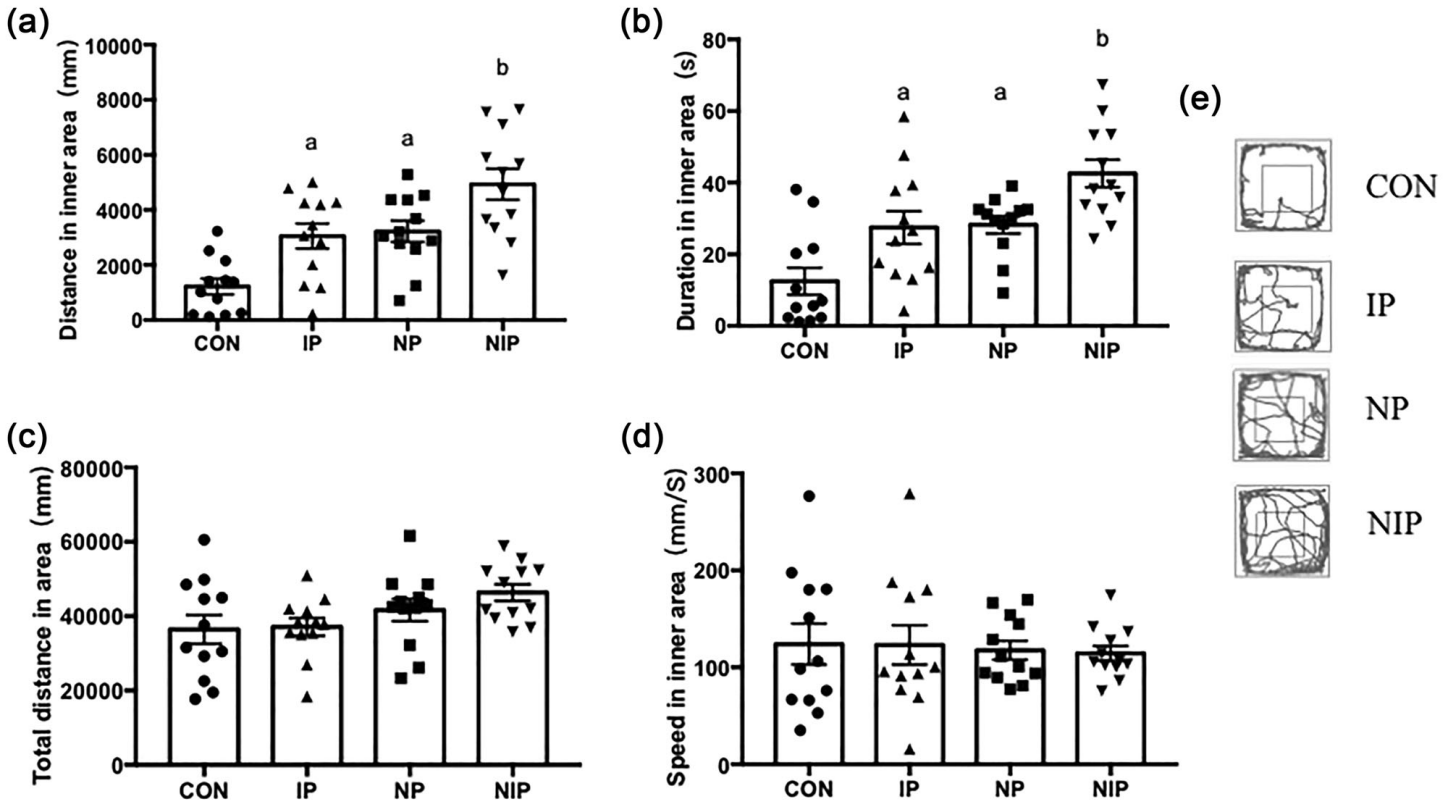
Open field performance: (a) distance traveled in the inner area; (b) time spent in inner area; (c) total distance traveled in the open field; (d) velocity in the inner area; (e) the motive paths in the open field test (OFT). ^a^ *p* < .05, different with the control (CON) group, ^b^ *p* < .05, different from all other groups, two‐way ANOVA with Tukey post hoc comparison. Data are presented as mean ± SEM, *n* = 12 per group.

We conducted the OFT to assess movement and response to anxiety‐inducing stimuli. As shown in Figure 3, the distance (*F*[3, 44] = 12.17, *p* < .0001) and time (*F*[3, 44] = 10.78, *p* < .0001) in the inner area showed significant differences among the four groups of rats (Figure 3a,b). Both the IP and NP groups exhibited increased activity time and distance in the inner area compared with the CON group (*p* < .05), whereas the NIP group showed higher time and distance than all the other three groups (*p* < .05). Nevertheless, no difference was observed in either total distance (*F*[3, 44] = 2.44, *p* > .05, Figure 3c) or speed in the inner area (*F*[3, 44] = 0.08, *p* > .05, Figure 3d) between the four groups.

### 3.5 Mixed pain exacerbates the impairment of fear memory

**FIGURE 4:**
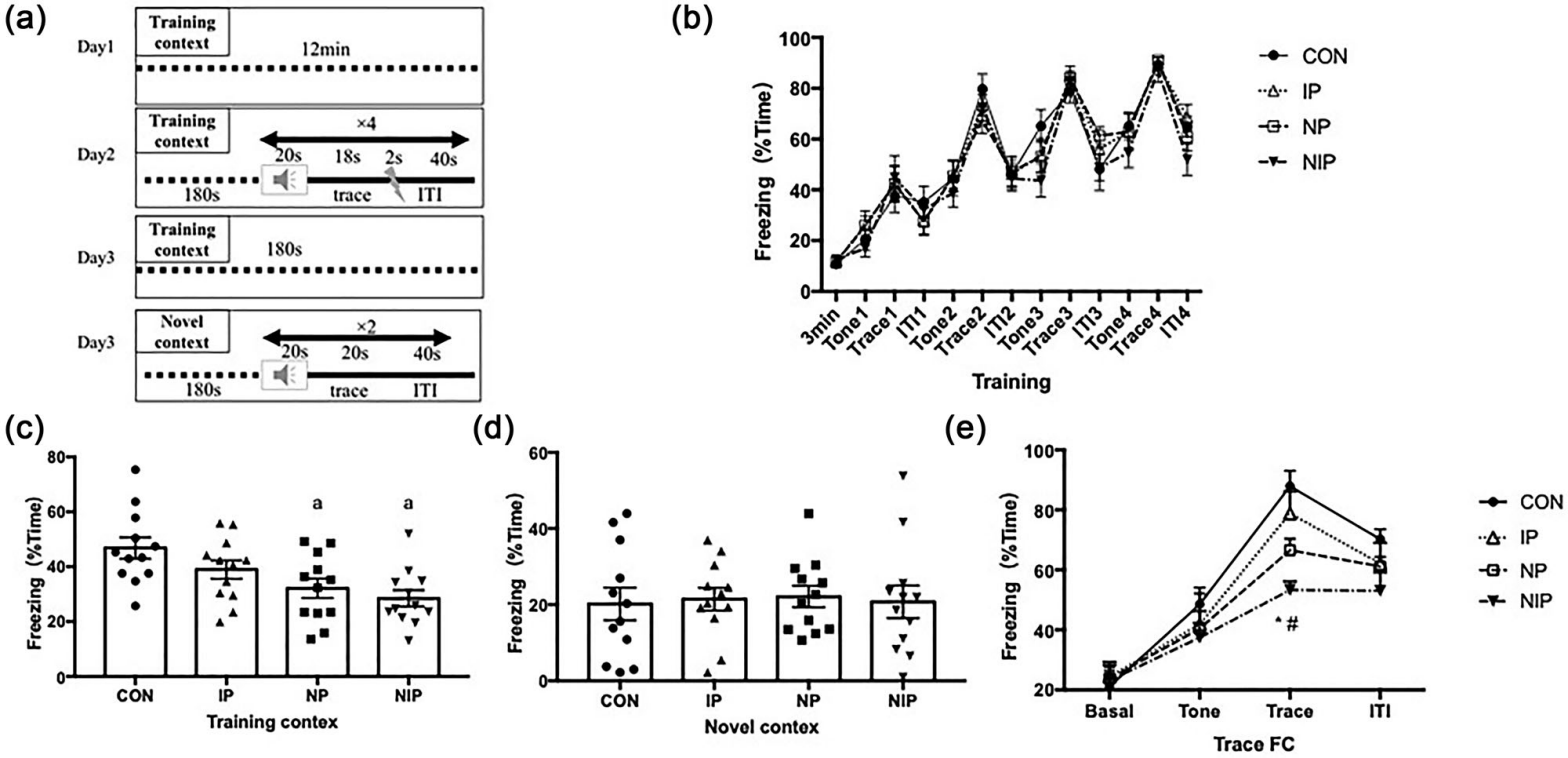
Trace fear conditioning (FC) performance: (a) experimental protocol; (b) freezing behavior during the training phase; (c) freezing behavior in the training chamber during contextual FC. ^a^ *p* < .05, compared with the groups of control (CON) and inflammatory pain (IP), one‐way ANOVA with Tukey post hoc comparison. (d) Freezing behavior in the novel chamber during contextual FC; (e) freezing performance without any shocks during trace FC phase. ^*^The percentage of freezing time in the needling pain (NP) group was lower than those in the CON and IP groups, *p* < .05. ^#^The percentage of freezing time in needling and inflammatory pain (NIP) group was lower than those in CON, IP, and NP group, *p* < .05, two‐way ANOVA with Tukey post hoc comparison. Data were expressed as mean ± SEM, *n* = 12. ITI, intertrial interval.

The trace FC test was performed to assess emotion‐related memory (Figure 4a). On the second day of the training session, we observed a gradual increase in immobility in all groups. There was no difference in the behavior trajectory among the four groups (*F*[3, 572] = 1.89, *p* > .05, Figure 4b), indicating that the rats in all groups could acquire fear memory of shock immediately after conditioned training.

On the third day in the test phase, when the rat pups reentered the training box, there was a significant difference in the freezing time among the four groups (*F*[3, 44] = 5.45, *p* < .01, Figure 4c). NP and NIP groups showed a significant reduction of freezing time compared to the control group (*p* < .05). There was no significant difference between the IP and the other three groups (*p* > .05). However, there was no difference in freezing time among the groups after changing the background (*F*[3, 44] = 0.05, *p* > .05, Figure 4d). The trace FC behavior in the changed context was examined on the third day. As shown in Figure 4e, effect of pain on freezing time was significant in the trace phase (*F*[3, 176] = 11.53, *p* < .0001). The freezing time of trace FC in the NP group was significantly lower than that in the CON group (*p* < .05). The NIP group showed a greater decrease in freezing time of trace FC than the other three groups (*p* < .05).

### 3.6 Mixed pain caused promotes secretion of corticosterone into the blood serum

**FIGURE 5:**
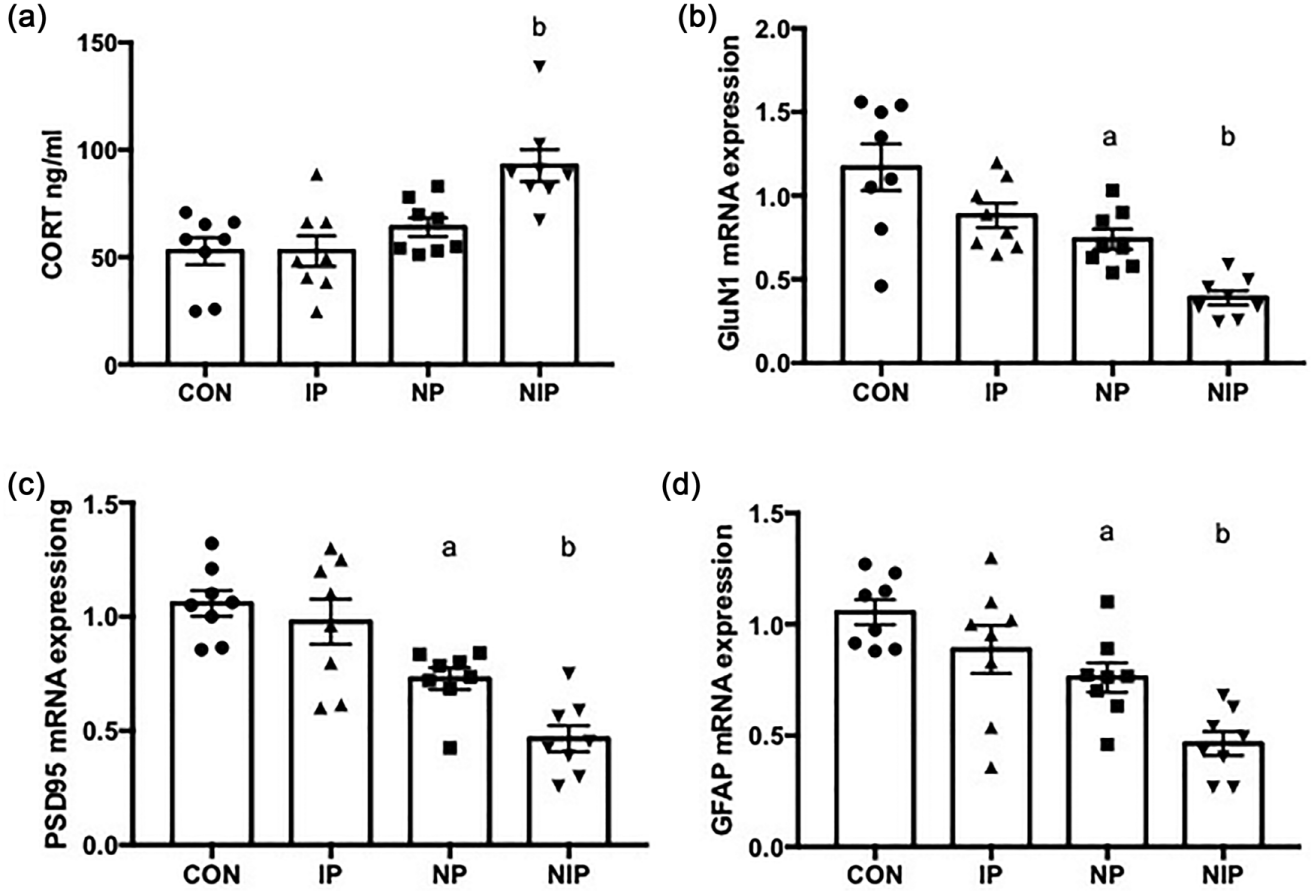
The expression levels of corticosterone (CORT) in the serum of rats (a). The mRNA expression of molecules involved in synaptic development in medial prefrontal cortex (mPFC) (b–d): GluN1 (b), postsynaptic density protein 95 (PSD95) (c), glial fibrillary acidic protein (GFAP) (d). ^a^ *p* < .05, compared to the control (CON) group, ^b^ *p* < .05, different from all other groups, one‐way ANOVA with Tukey post hoc comparison. Data are expressed as the mean ± SEM, *n* = 8 per group.

Peripheral blood serum was collected 30 min after the test of trace FC, and serum corticosterone was detected to observe the response of rats to stresses after behavioral experiments. As shown in Figure 5a, there was a significant difference in corticosterone secretion of the four groups (*F*[3, 28] = 8.601, *p* < .001). Among them, NIP group showed a higher corticosterone in comparison to the remaining three groups (*p* < .05), and there was no difference in corticosterone level among the other three groups (*p* > .05).

### 3.7 Mixed pain downregulates synapses and astrocyte‐associated molecules in the mPFC

**FIGURE 6:**
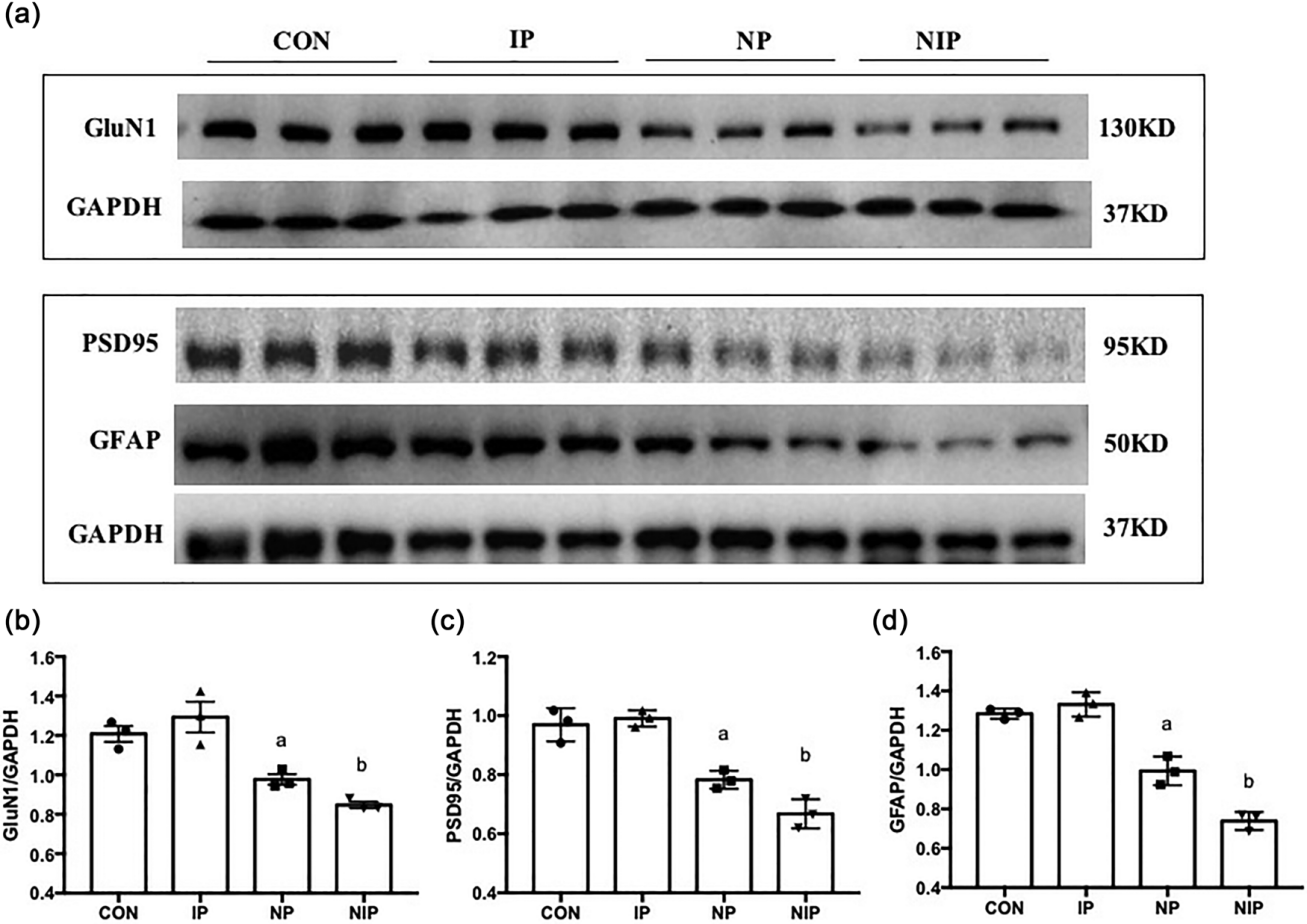
The protein levels of molecules involved in synaptic development in medial prefrontal cortex (mPFC): (a) representative blots of GluN1, postsynaptic density protein 95 (PSD95), glial fibrillary acidic protein (GFAP), and glyceraldehyde‐3‐phosphate dehydrogenase (GAPDH); (b) the relative density of GluN1 normalized to GAPDH; (c) the relative density of PSD95; (d) the relative density of GFAP. ^a^ *p* < .05, compared to the groups of control (CON) and inflammatory pain (IP), ^b^ *p* < .05, different from all other groups, one‐way ANOVA with Tukey post hoc comparison. The data are expressed as the mean ± SEM, *n* = 3 per group.

In order to analyze the effect of neonatal pain on synaptic development, we examined the mRNA level of GluN1, PSD95, and GFAP in whole mPFC tissue. As shown in Figure 5b–d, the expression levels of these three molecules were significantly different among the four groups of rats (GluN1 *F*[3, 28] = 13.91, *p* < .0001; PSD95 *F*[3, 28] = 15.52, *p* < .0001; GFAP *F*[3, 28] = 11.27, *p* < .0001). The mRNA levels of GluN1, PSD95, and GFAP in the mPFC decreased in the NP, compared to those in the CON group on PD26 (*p* < .05); those in the NIP group were lower than those in the CON, IP, and NP groups (*p* < .05). Similarly, as shown in Figure 6a–d, the protein level of GluN1, PSD95, and GFAP dropped in the NP group, compared to those in the CON group (*p* < .05); those in the NIP group were lower than those in the other three groups (*p* < .05).

## 4 DISCUSSION

Exposure to repetitive procedure pain leads to long‐term adverse effects, especially the development of somatic sensation and cognition (Butkevich et al., 2021; Williams & Lascelles, 2020). In this study, we compared the effects of three types of neonatal pain on somatosensory and cognitive development in later life. The finding revealed that neonatal NP significantly reduced pain threshold, impaired conditioned memory, caused insensitivity to anxiety‐causing stimuli, and decreased expression of some factors related to synaptic development. Moreover, the combination of needling pricks and substance induced IP in neonatal pubs may exert a more serious adverse effect on somatosensory and cognitive development, which suggests that the effective efforts should be taken to relieve pain in preterm infants, thus ensuring a normal neurodevelopment.

In the present study, needling was performed to simulate the pain from repeated skin punctures in NICU and carrageenan injection to induce the pain from inflammation. It has been confirmed that the concurrence of multiple perinatal stressors may accelerate demyelination, microglial activation, and dysregulate the expression of glial markers, like GFAP in the CNS (Sarkar et al., 2019). Moreover, the combination of multiple early adverse experiences may increase the risk of pain hypersensitivity in children (You et al., 2019). These data, together with ours, support that multiple painful stimuli in the neonatal period may exacerbate the adverse effects on somatosensory and cognitive behaviors.

In this study, neonatal NP significantly decreased the pain threshold of hindpaw in rats, but the sensitivity to pain episodes did not change significantly in the IP group. One previous research has compared the effects of low and high doses of carrageenan on MWT, finding that neither affected long‐term painful sensitivity (Walker et al., 2003). In the present study, we administered a low dose of carrageenan to rats and observed that carrageenan stimulation caused foot edema for approximately 24 h, but no long‐term alternation in mechanical sensitivity. These data indicated that the carrageenan injection, as an acute inflammatory stimulus, may not be serious enough to affect the long‐term mechanical sensitivity. The IP model can also be constructed by injecting complete Freund adjuvant (CFA), which often leads to severe chronic arthritis in the adult animals. CFA injection in the neonatal period may induce chronic foot inflammation that proceeds until adolescence (Walker et al., 2003) and result in a lower pain threshold (Roizenblatt et al., 2010). These previous findings suggest that CFA injection in neonates is more likely to cause chronic inflammation and abnormal mechanical sensitivity that may persist into adulthood. In the present study, however, carrageenan injection just simulated transient neonatal inflammation and did not affect the pain sensitivity and memory of rats after weaning. The combination of NIP within PD7 decreased the mechanical and thermal pain thresholds after weaning, which was similar to the effect in the NP group. However, upon secondary inflammatory stimulation at PD21, the NIP group showed a higher duration of hypersensitivity to pain than the NP group, suggesting that neonatal mixed pain would cause a more serious response after a second stimulation.

Pain can activate the hypothalamic‐pituitary‐adrenal (HPA) axis to induce corticosterone release. Some previous studies have examined the long‐term impact of early‐life pain on mediators of stress reactivity. Victoria et al. (2014) found a notable surge in serum corticosterone at 24 h and 7 days of rats after receiving a single IP on PD0. Another study has revealed no difference in corticosterone before stress but significant downregulation of corticosterone following restraint or swim stress in adult rats with neonatal painful experience (Victoria et al., 2013a), suggesting a potentially permanent effect on glucocorticoid system of early‐life stress. In this study, neither repetitive NP nor IP in the neonatal period dysregulated corticosterone secretion in juvenile rats, which is consistent with some previous researches (Anand et al., 1999; Davis et al., 2018), but different from the discovery of Victoria et al. One explanation may be that the corticosterone is detected under different stress conditions. We tested the corticosterone secretion half an hour after the test phase of trace FC, a time point under no strong stimuli, whereas restrictive and swimming stress in the study of Victoria may result in a more potent response.

Interestingly, neonatal mixed pain in the NIP group could increase corticosterone secretion significantly at PD26, which may be associated with the longer duration of high sensitivity to pain after the second stimulation. It has been confirmed that greater cortisol release suggests a higher sensitivity to pain (Lu et al., 2013). In addition, the thicker hindpaw in the NIP group in neonatal time may indicate a stronger local inflammatory response that exceeds the regulatory threshold of the sensory system and poses in a plastic effect on sensory pathways.

Either repetitive NP or single IP resulted in insensitivity to anxiety‐causing stimuli in OFT, which is consistent with some previous studies (Chen et al., 2016; Victoria et al., 2013b). However, de Kort et al. (2021) achieved a different finding that neonatal four needle pricks daily in the left hindpaw did not affect the results of the OFT but increased the activity time in open arm of the elevated zero maze, which is also a manifestation of insensitivity to anxiety‐causing stimuli. The activity time in the open arm of the elevated plus maze also increased in juvenile rats having received neonatal four needling pricks daily in the left hindpaw (Zuke et al., 2019). Nevertheless, the activity time in both OFT and elevated plus maze showed no difference in the neonatal rats with ten needle pricks per day (Ranger et al., 2018). The elevated zero maze and elevated plus maze mainly represent state anxiety, whereas the open field refers to trait anxiety. The inconsistent performances may stem from differences in number and location of needle prick in the neonatal pain model.

Multiple studies have focused on the cognitive outcomes of one single type of pain exposure. Consistent with other results (Davis et al., 2018; Nuseir et al., 2017; Ranger et al., 2018; Xia et al., 2020), we confirmed that repeated NP rather than single inflammatory stimulus, impaired the conditioned memory. More importantly here, mixed painful stimulus worsens cognitive impairments. This effect is likely due to neonatal trauma‐interrupted fear conditioning or fear expression to the context and auditory cues. A study revealed that early‐life stress decreases the growth of synaptic dendrite spines and leads to the maladaptation of neurons to stress (Cao et al., 2021). The deficits in cognitive function in this study following neonatal pain may also be related with previous findings of altered dendritic spines associated with early‐life stress.

Cognition arises from synaptic transmission in the mPFC, which involves various structural molecules and membrane receptors. Previous studies have shown that early‐life stress impairs synaptic function. For example, neonatal maternal separation may be followed by a low level of PSD95 (Marco et al., 2013), a vital marker of synapses in the posterior membrane (Fitzgerald et al., 2015). We have also demonstrated that neonatal painful stimulation decreases the expression of PSD95 and GluN1, a protein implicated in synaptic transmission and cognitive development (Glen et al., 2014; Zhou & Duan, 2021). In addition, mixed pain in the neonatal period further exacerbates the impairment of cognitive behaviors and decline of synapse‐related molecules, which may also be associated with corticosterone oversecretion that may cause neuroexcitotoxicity and synaptic defects (Holtzman et al., 2013).

Astrocytes are involved in glutamate transport to prevent the excitotoxic effect caused by excess glutamate on neuron and synapses after stress (Blanco‐Suárez et al., 2017). Specific proteins produced in the glial cells are used as markers to identify glial stimulation and genesis, and the effect of early‐life stressors on glial cells can be studied through the upregulation or downregulation of such specific markers (Sarkar et al., 2019). The GFAP, a molecular marker of astrocytes, changes its expression upon stressful events (Zhang et al., 2017). We found for the first time that repetitive NP led to decreased level of GFAP expression. Abnormal GFAP expression may affect synaptic development and cognitive function (Xie et al., 2022). Stress caused by early maternal separation also downregulates GFAP and retards cognitive development (Yamawaki et al., 2018). These findings reveal that early‐life stress can affects the long‐term functional plasticity of astrocytes and synaptic development, which may be associated with the decreased neurotrophic effect or glutamate transport (Çalışkan et al., 2020).

## 5 CONCLUSION

This study revealed that a combination of NIP exerts more serious effect on neonates’ neurodevelopment through increasing the pain hypersensitivity, impairing conditional memory, inducing the insensitivity to anxiety‐causing stimuli, and damaging synaptic function in the mPFC. Efforts should be taken to relieve pain in preterm infants.

## 6 LIMITATION

In this study, we did not detect serum cortisol levels immediately after model establishment. The degree of inflammation was just determined by the thickness of the hindpaw, and different modeling methods may cause various degrees of stress response. Corticosterone levels at different time points after secondary stimulation, which may associate with the mechanical threshold, were not tested. Previous study showed that the chronic stress induces vulnerability to perturbations and dysregulation of HPA activity in adult rats with neonatal pain, indicating that the rats may experience changes in their behavior after repeated instances of stress (Victoria et al., 2015). In this study, we used the same animals in several behavioral experiments, which are also some unpredictable stressors and could affect behaviors when faced with the subsequent challenge and expression of related molecules. More attention should be paid to the mechanism under which the animals exposed to neonatal pain demonstrate an altered behavioral profile when challenged with multiple stressors.

## AUTHOR CONTRIBUTIONS

Ru Ling, Cuiting Min, and Xiaonan Li participated in conception, design the research, and interpreted results of experiments; Yueshu Wang and Wen Zheng performed the behavioral tests; Mengying Chen and Dongqing Xia contributed to biochemical analysis. Ru Ling and Xiaonan Li drafted the manuscript. All authors read and revised the manuscript and approved the final version.

## CONFLICT OF INTEREST STATEMENT

All authors claim that there are no conflicts of interest.

### PEER REVIEW

The peer review history for this article is available at https://publons.com/publon/10.1002/brb3.3309.

## Supporting information

Supporting Information 1 Left hindpaw thickness measurements of neonatal rat pups at PD7. Data are shown as mean ± SEM. One‐way ANOVA with Tukey post hoc comparison. *n* = 12 per group.

## Data Availability

The data that support the findings of this study are available from the corresponding author upon reasonable request.
