# Supplementary figures and images for: Effects of different types of neonatal pain on somatosensory and cognitive development in male juvenile rats
Source: Brain Behav. 2023 Nov 15;13(12):e3309. doi: 10.1002/brb3.3309 (PMC10726798; doi:10.1002/brb3.3309)

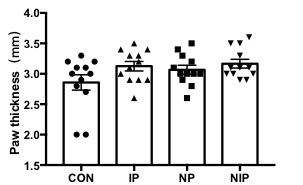

Supplement: Supplementary file 1 — Supporting Information 1 Left hindpaw thickness measurements of neonatal rat pups at PD7. Data are shown as mean ± SEM. One‐way ANOVA with Tukey post hoc comparison. n = 12 per group. [file BRB3-13-e3309-s001.jpg]
